# Einmal geimpft, immer geimpft? Routinedatenanalyse zur wiederholten Influenzaimpfung von ≥ 60-Jährigen

**DOI:** 10.1007/s00103-023-03716-1

**Published:** 2023-06-08

**Authors:** Josephine Storch, Franziska Meissner, Monique Böde, Anja Kwetkat, Mathias W. Pletz, Antje Freytag

**Affiliations:** 1 Universitätsklinikum Jena, Institut für Allgemeinmedizin, Friedrich-Schiller-Universität, Bachstr. 18, 07743 Jena, Deutschland; 2 Internationale Graduierten Akademie, Medizinische Fakultät, Institut für Gesundheits- und Pflegewissenschaft, Martin-Luther-Universität Halle-Wittenberg, Halle (Saale), Deutschland; 3 Klinik für Geriatrie und Palliativmedizin, Klinikum Osnabrück, Osnabrück, Deutschland; 4 Universitätsklinikum Jena, Institut für Infektionsmedizin und Krankenhaushygiene, Friedrich-Schiller-Universität, Jena, Deutschland

**Keywords:** Beobachtungsstudie, Influenza, Ältere, Primärversorgung, Impfempfehlung, Observational study, Influenza, Elderly, Primary care, Vaccination recommendation

## Abstract

**Hintergrund:**

Die Ständige Impfkommission empfiehlt eine jährliche Influenzaimpfung als Standardimpfung für ≥ 60-Jährige und als altersunabhängige Indikationsimpfung. Empirische Daten zur wiederholten Impfung liegen für Deutschland nicht vor. Ziel der Studie war es daher, Häufigkeit und Einflussfaktoren der wiederholten Impfung zu untersuchen.

**Methoden:**

Wir führten eine längsschnittliche retrospektive Beobachtungsstudie mit Routinedaten von ≥ 60‑jährigen Versicherten der Thüringer AOK Plus im Zeitraum 2012–2018 durch. Die Anzahl der Saisons mit Impfung wurde beschrieben und der Zusammenhang mit verschiedenen Versichertenmerkmalen in einem Regressionsmodell analysiert.

**Ergebnisse:**

Es wurden 103.163 Versicherte mit mindestens einer Impfung in der Saison 2014/2015 eingeschlossen, von denen 75,3 % in ≥ 6 von 7 Saisons geimpft wurden. Häufigere Impfungen zeigten sich bei Pflegeheimbewohner:innen (Rate Ratio (RR) 1,27), Personen mit erhöhter gesundheitlicher Gefährdung infolge einer Grunderkrankung (RR 1,21) und höheren Altersgruppen (vs. 60- bis 69-Jährige: RR 1,17–1,25). Mit jedem zusätzlichen Jahr der Teilnahme an einem Disease-Management-Programm stieg die Anzahl der Impfungen (RR 1,03). Weniger häufig geimpft waren Frauen (RR 0,91), Versicherte mit Pflegestufe 1 (vs. keiner Pflegestufe: RR 0,90) und Versicherte mit einer Komorbidität (vs. keiner Komorbidität: RR 0,97).

**Diskussion:**

Ein Großteil der einmal gegen Influenza geimpften ≥ 60-Jährigen lässt sich auch wiederholt impfen. Entsprechend den Empfehlungen sind vor allem Pflegeheimbewohner:innen und Personen mit erhöhter gesundheitlicher Gefährdung wiederholt geimpft. Hausärzt:innen kommt eine zentrale Rolle zu: Nichtakute Patientenkontakte sollten für Impfangebote genutzt werden, insbesondere bei Frauen und in der Häuslichkeit lebenden Pflegebedürftigen.

**Zusatzmaterial online:**

Zusätzliche Informationen sind in der Online-Version dieses Artikels (10.1007/s00103-023-03716-1) enthalten.

## Abstract (en)

**Background:**

The Standing Committee on Vaccination recommends the seasonal influenza vaccination as a standard vaccination for individuals aged ≥ 60 years and as an indication vaccination independent of age. Empirical data on repeated vaccination are not available for Germany. The aim of this study was therefore to investigate the frequency and influencing factors of repeated vaccinations.

**Methods:**

We conducted a longitudinal retrospective observational study with claims data from ≥ 60‑year-olds insured with the statutory health insurance AOK Plus in Thuringia between 2012 and 2018. The number of seasons with influenza vaccination was described and the association with various individual characteristics was analysed in a regression model.

**Results:**

Included were 103,163 individuals with at least one influenza vaccination in the 2014/2015 season, of whom 75.3% had been vaccinated in ≥ 6 of 7 seasons. We found repeated vaccinations more frequently among nursing home residents (rate ratio (RR) 1.27), individuals with increased health risk due to underlying diseases (RR 1.21) and higher age groups (vs. 60–69 years: RR 1.17–1.25). With each additional year of participating in a disease management program, the number of vaccinations increased (RR 1.03). Women (RR 0.91), individuals with nursing care level 1 (vs. no nursing care level: RR 0.90) and people with a comorbidity (vs. no comorbidity: RR 0.97) were less likely to receive repeated vaccinations.

**Discussion:**

A large proportion of individuals aged ≥ 60 years who have been vaccinated against influenza once is likely to repeatedly receive vaccinations. In accordance with vaccination recommendations, nursing home residents and in particular individuals with an increased health risk are vaccinated repeatedly. General practitioners play a central role: non-acute patient contacts should be used to offer vaccinations, especially to women and individuals in need of care who are living at home.

## Hintergrund

Influenza ist eine Infektionserkrankung der Atemwege, die jährlich ca. 1–9 Mio. zusätzliche Arztkonsultationen in Deutschland verursacht [1, 2] und vor allem bei ≥ 60-Jährigen und weiteren Risikogruppen zu schweren Krankheitsverläufen und Todesfällen führen kann [3, 4]. So kam es in den Saisons 2014/2015 und 2016/2017 insbesondere bei ≥ 60-Jährigen zu hohen Hospitalisierungsraten und überdurchschnittlich vielen Todesfällen [5, 6]. Daher empfiehlt die Ständige Impfkommission (STIKO) beim Robert Koch-Institut (RKI) eine jährliche Influenzaimpfung mit einem saisonalen Impfstoff als wichtige Präventionsmaßnahme. Die Impfung wird für alle ≥ 60-Jährigen als Standardimpfung sowie, unabhängig vom Alter, bei Vorliegen bestimmter Grunderkrankungen und für Pflegeheimbewohner:innen als Indikationsimpfung empfohlen [7].

Seit der Influenzasaison 2014/2015 erfolgt das stetige Impfmonitoring im Rahmen der KV-Impfsurveillance am RKI. Hier werden anhand von Abrechnungsdaten der 17 deutschen kassenärztlichen Vereinigungen (KV) die Impfquoten bei Versicherten der gesetzlichen Krankenversicherung (GKV) für jede Saison (definiert als Zeitraum vom dritten Quartal eines Jahres bis inklusive des ersten Quartals des Folgejahres, Q341) im Querschnitt erhoben und auf die Gesamtbevölkerung hochgerechnet [8, 9]. Längsschnitterhebungen, also individuumsbezogene Analysen, zur Inanspruchnahme wiederholter Influenzaimpfungen über mehrere Jahre bei ≥ 60-Jährigen sind darin nicht enthalten und auch insgesamt rar. So gibt es nur wenige internationale Studien, die dies untersucht haben [10–13]. Für Deutschland fand sich nur eine Studie, in der die wiederholte Influenzaimpfung über 3 Saisons als ein Teilergebnis berichtet wurde [14]. Zahlen zum individuellen Influenzaimpfverhalten Älterer über einen längeren Zeitraum liegen nach bisherigem Wissensstand nicht vor. Eine empirische Überprüfung der wiederholten Impfung ist relevant, da mit zunehmendem Alter die Immunantwort nachlässt (Immunseneszenz) und die Krankheitslast durch Influenza steigt. Zudem ist die Impfung jährlich mit einem angepassten Impfstoff empfohlen, da die Erregereigenschaften aufgrund von Mutationsmechanismen verändert sein können und die Zusammensetzung der zirkulierenden Virussubtypen variieren kann. Zu wissen, wer sich wiederholt impfen lässt und welche Faktoren einen Einfluss darauf haben, kann helfen, Personen gezielt anzusprechen, sie in ihrer Impfentscheidung zu unterstützen und so die Impfquoten zu erhöhen.

Ziel der Arbeit war es daher, die Inanspruchnahme der wiederholten Influenzaimpfung von ≥ 60-Jährigen anhand von GKV-Abrechnungsdaten zu untersuchen und dabei Merkmale von Versicherten zu identifizieren, die mit einer wiederholten Influenzaimpfung in Zusammenhang stehen.

## Methoden

### Studiendesign, Datengrundlage und Studienpopulation

Wir führten eine retrospektive, längsschnittliche Beobachtungsstudie über die Jahre 2012–2018 als Sekundäranalyse durch. Datengrundlage bildeten die Abrechnungsdaten der AOK Plus zu Thüringer Versicherten ≥ 60 Jahre aus dem vom Bundesministerium für Bildung und Forschung geförderten Verbundprojekt „Impfen60+ – Impfbereitschaft 60+ fördern“ (Förderkennzeichen: 03ZZ0819B; [15]). Diese Abrechnungsdaten dienten in der Primärstudie der gesundheitsökonomischen Evaluation der Influenza- und Pneumokokkenimpfungen [16] sowie der Analyse der Impfeffekte hinsichtlich der Krankheitslast [17] und unterlagen daher definierten Einschlusskriterien (siehe Onlinematerial).

Die AOK Plus ist die größte GKV in Thüringen und deckt über 50 % der gesetzlich Versicherten ab [18]. Es lagen Abrechnungsdaten aus allen ambulanten und stationären Leistungsbereichen für die Jahre 2012–2018 vor. Anhand einer individuellen pseudonymisierten Identifikationsnummer konnten die in Anspruch genommenen Leistungen einem bzw. einer Versicherten über den gesamten Beobachtungszeitraum zugeordnet werden. Die Identifikation der Impfungen erfolgte anhand aller relevanten Gebührenordnungspositionen des Einheitlichen Bewertungsmaßstabs (siehe Onlinematerial Tabelle A1). In Design, Durchführung und Bericht der Studie folgen wir den Empfehlungen der Standardisierten Berichtsroutine für Sekundärdaten Analysen (STROSA; [19]).

Ausgangspopulation der Sekundäranalyse bildeten die Daten von 142.022 Versicherten (für eine Abgrenzung zur Studienpopulation der Primärstudie, siehe Onlinematerial Abb. A1). Hiervon wurden nur Versicherte eingeschlossen, die 2014Q34 eine Influenzaimpfung erhalten hatten. Weitere Einschlusskriterien waren: mindestens 60 Jahre zum 01.01.2012, durchgängig bei der AOK Plus versichert, Wohnsitz in Thüringen im Zeitraum 2012–2018. Ausgeschlossen wurden Versicherte, die im Beobachtungszeitraum verstarben oder keine Influenzaimpfung bzw. nur eine Pneumokokkenimpfung erhalten hatten.

### Datenaufbereitung

Die Anzahl der Saisons mit Influenzaimpfungen wurde im Zeitraum 2012–2018 gemessen. Ein Versicherter bzw. eine Versicherte galt in einer Saison als geimpft, wenn in Q341 mindestens eine Influenzaimpfung abgerechnet wurde (siehe Onlinematerial Tab. A1). Ausnahme bildet die Saison 2018Q34, da die Daten für 2019 nicht vorlagen und somit 2019Q1 nicht berücksichtigt werden konnte. Zur besseren Lesbarkeit wird das Outcome im Folgenden verkürzt als „Anzahl der Influenzaimpfungen“ benannt.

Auf Basis der STIKO-Empfehlung [7], einer explorativen Literaturrecherche sowie Expertenmeinungen im Bereich der Geriatrie und Infektiologie wurden in GKV-Abrechnungsdaten enthaltene Versichertenmerkmale identifiziert, die im Zusammenhang mit der Anzahl der Influenzaimpfungen stehen können. Eines dieser Merkmale ist die Altersgruppe (zum 01.01.2012), da Ältere sich häufiger gegen Influenza impfen lassen [20] bzw. das Alter in Zusammenhang mit der Regelmäßigkeit der Impfung steht [10]. Ein weiteres Merkmal ist das Geschlecht, da Frauen sich laut Studien häufiger und regelmäßiger impfen lassen [10, 21, 22].

Die bestehende Impfempfehlung für Pflegeheimbewohner:innen und die Erkenntnis, dass mit Einzug ins Pflegeheim auch die Impfquoten steigen [23], ließen die Annahme zu, dass Pflegebedürftige sich grundsätzlich häufiger impfen lassen. Daher wurden als mögliche Prädiktoren die Pflegestufe (PflS), die aufgrund fehlender Daten für die Folgejahre als Stichtagsangabe zum 31.12.2013 Berücksichtigung fand, sowie der Pflegeheimaufenthalt im Zeitraum 2012–2018 einbezogen. Eine versicherte Person galt dann als Pflegeheimbewohner:in, wenn in jedem Jahr mindestens ein Pflegeheimaufenthalt vorlag. Versicherte, die im gesamten Zeitraum 2012–2018 keinen Pflegeheimaufenthalt hatten, wurden als durchgängig nicht im Pflegeheim lebend gekennzeichnet. Um eine Reduktion der Teststärke durch Fallausschluss zu verhindern und im Regressionsmodell die gesamte Studienpopulation betrachten zu können, wurden Versicherte, die weder durchgängig im Pflegeheim noch durchgängig in der Häuslichkeit lebten, einer dritten Gruppe („Mischform“) zugewiesen. Die Heterogenität dieser dritten Gruppe verhindert eine klare Interpretation ihrer Ergebnisse, weshalb auf diese nicht explizit eingegangen wird.

Unter der Annahme, dass Versicherte mit regelmäßigen ärztlichen Kontakten häufiger geimpft werden, wurde zur Abbildung der Versorgungskontinuität die Anzahl der Jahre mit Einschreibung in ein Disease-Management-Programm (DMP) im Zeitraum 2012–2018 herangezogen, da hierin regelmäßige Vorstellungen in der Arztpraxis erforderlich sind.

Zur Berücksichtigung impfrelevanter Grunderkrankungen wurden Personen mit erhöhter gesundheitlicher Gefährdung identifiziert. Dies traf dann zu, wenn im Jahr 2013 mindestens eine primäre oder sekundäre Krankenhausentlassungsdiagnose oder gesicherte ambulante Diagnose nach der 10. Version der internationalen statistischen Klassifikation der Krankheiten und verwandter Gesundheitsprobleme (ICD-10-GM) codiert war, die gemäß der Influenzaimpfempfehlung einer Erkrankungsgruppe für eine Indikationsimpfung zugeordnet werden konnte ([24]; siehe Onlinematerial Tab. A2). Zusätzlich wurde die Summe der Charlson-Komorbiditäten des Charlson Comorbidity Index (CCI) nach der Definition von Schwarzkopf et al. [25] ermittelt (siehe Onlinematerial Tab. A3). Diese können mit einer häufigeren Impfung einhergehen [26, 27] und wurden ebenfalls anhand des Vorliegens von mindestens einer primären oder sekundären Krankenhausentlassungsdiagnose oder gesicherten ambulanten Diagnose nach ICD-10-GM im Jahr 2013 identifiziert.

Um zu eruieren, welche Arztgruppen an der Impfung beteiligt sind und ob Versicherte, die sich häufiger impfen lassen, dies dann auch in derselben Arztpraxis tun, wurde zudem die Anzahl der Influenzaimpfungen durch Hausärzt:innen sowie durch dieselbe Arztpraxis beschrieben. Als Hausärzt:in galten Fachärzt:innen für Allgemeinmedizin, hausärztliche praktische Ärzt:innen ohne Facharztweiterbildung und Fachärzt:innen für hausärztliche innere Medizin. Alle anderen Arztgruppen wurden Fachärzt:innen zugeordnet. Die Prüfung und Aufbereitung der Daten erfolgte in SAS, Version 9.4 (SAS Institute Inc., Cary, NC, USA).

### Statistische Analysen

Zur Analyse des Zusammenhangs zwischen Einflussfaktoren und der Häufigkeit der Influenzaimpfungen im Zeitraum 2012–2018 wurde ein multiples negativ-binomiales Regressionsmodell angewendet. Aufgrund der linksschiefen Verteilung der Anzahl der Influenzaimpfungen wurde das Outcome für diese Analyse invertiert. Die so berechneten Regressionskoeffizienten wurden schließlich erneut invertiert, um die Rate Ratios (RR) und dazugehörigen Konfidenzintervalle (KI) entsprechend der ursprünglichen Polung des Outcomes interpretieren zu können. Zur Überprüfung der Annahme, dass mit steigender PflS auch die Anzahl an Influenzaimpfungen steigt, ging die PflS als 4‑stufiger kategorialer Prädiktor mit *Backward Difference Coding* in das Regressionsmodell ein. Der Effekt der PflS kann so mit 3 Kontrasten beschrieben werden, die jeweils 2 aufeinanderfolgende PflS miteinander vergleichen (PflS 1 (inkl. PflS 0) vs. keine PflS, PflS 2 vs. PflS 1, PflS 3 (inkl. Härtefälle) vs. PflS 2). Vor dem Hintergrund, dass der Effekt der PflS, je nachdem ob eine versicherte Person im Pflegeheim lebte oder nicht, unterschiedlich ausfallen könnte, wurde das Modell in einem zweiten Schritt um die Interaktion von Pflegeheim und PflS erweitert. Es zeigte sich, dass sich der Effekt der PflS auf die Zahl der Impfungen zwischen Pflegeheim- und Nicht-Pflegeheimbewohner:innen nicht statistisch signifikant unterscheidet (*p* ≥ 0,380 für alle Interaktionskontraste). Daher wird aus Gründen der Sparsamkeit das Modell ohne Interaktion berichtet. Die Analysen erfolgten in R, Version 4.1.2 (The R Foundation for Statistical Computing).

## Ergebnisse

### Studienpopulation

Insgesamt konnten 103.163 Versicherte in die Studienpopulation eingeschlossen werden, die mindestens einmal (d. h. in 2014Q34) gegen Influenza geimpft worden waren. Diese waren im Durchschnitt 72,6 Jahre alt und zu 61,1 % (*n* *=* 63.036) weiblich (Tab. 1). Im Jahr 2013 hatten 90,9 % (*n* *=* 93.823) der Versicherten keine PflS und 9,1 % mindestens PflS 1 (inkl. PflS 0, *n* *=* 6678; Tab. 1). Im Zeitraum 2012–2018 lebten 2,0 % (*n* *=* 2038) der Versicherten durchgängig im Pflegeheim und 90,0 % (*n* *=* 92.836) durchgängig nicht im Pflegeheim; 63,7 % (*n* *=* 65.708) der Versicherten nahmen an mindestens einem DMP teil (mit durchschnittlich 1,7-jähriger Einschreibedauer). Bei 93,1 % (*n* *=* 96.027) der Versicherten lag mindestens eine Erkrankung vor, nach der sie als Person mit erhöhter gesundheitlicher Gefährdung identifiziert werden konnten. 21,2 % (*n* *=* 21.862) der Versicherten hatten keine Komorbiditäten und mit 43,7 % hatten die meisten Versicherten (*n* *=* 45.120) 2–4 CCI-Komorbiditäten.

**Table 1:** 

	Gesamt (*N* = 103.163)	
*Alter*		
*Alter in Jahren*, MW (SD)	72,6	(7,0)
*Altersgruppe, n* (%)		
60–69 Jahre	33.689	(32,7 %)
70–79 Jahre	51.485	(49,9 %)
80–89 Jahre	17.315	(16,8 %)
≥ 90 Jahre	674	(0,7 %)
*Geschlecht, n* (%)		
Männlich	40.127	(38,9 %)
Weiblich	63.036	(61,1 %)
*Pflegestufe zum 31.12.2013, n* (%)		
Keine Pflegestufe	93.823	(90,9 %)
Pflegestufe 1 (inkl. Pflegestufe 0)	6678	(6,5 %)
Pflegestufe 2	2224	(2,2 %)
Pflegestufe 3 (inkl. Härtefälle)	438	(0,4 %)
*Pflegeheim im Zeitraum 2012–2018, n* (%)		
Durchgängig nicht im Pflegeheim lebend	92.836	(90,0 %)
Durchgängig im Pflegeheim lebend	2038	(2,0 %)
Mischform	8289	(8,0 %)
*DMP-Teilnahme im Zeitraum 2012–2018*		
Teilnahme an mind. 1 DMP, *n* (%)	65.708	(63,7 %)
Jahre mit DMP-Teilnahme, MW (SD)	1,7	(1,9)
*Personen mit erhöhter gesundheitlicher Gefährdung im Jahr 2013, n* (%)		
*Personen mit erhöhter gesundheitlicher Gefährdung*	96.027	(93,1 %)
Chronische Immunsuppression	18.580	(18,0 %)
Herzerkrankung	61.099	(59,2 %)
Lungenerkrankung	25.405	(24,6 %)
Neurologische Erkrankung	27.707	(26,9 %)
Nierenerkrankung	20.962	(20,3 %)
Stoffwechselerkrankung	80.659	(78,2 %)
*Summe der Charlson-Komorbiditäten des Charlson Comorbidity Index (CCI) im Jahr 2013, n* (%)		
CCI: 0	21.862	(21,2 %)
CCI: 1	26.060	(25,3 %)
CCI: 2–4	45.120	(43,7 %)
CCI: ≥ 5	10.121	(9,8 %)
*Anzahl der Influenzaimpfungen pro Patient:in, abgerechnet durch* …, MW (SD)		
Einen Hausarzt/eine Hausärztin	5,9	(1,5)
Dieselbe Praxis	5,5	(1,6)

*DMP* Disease-Management-Programm, *MW* Mittelwert, *SD* Standardabweichung

### Anzahl der Influenzaimpfungen

**Figure 1:**
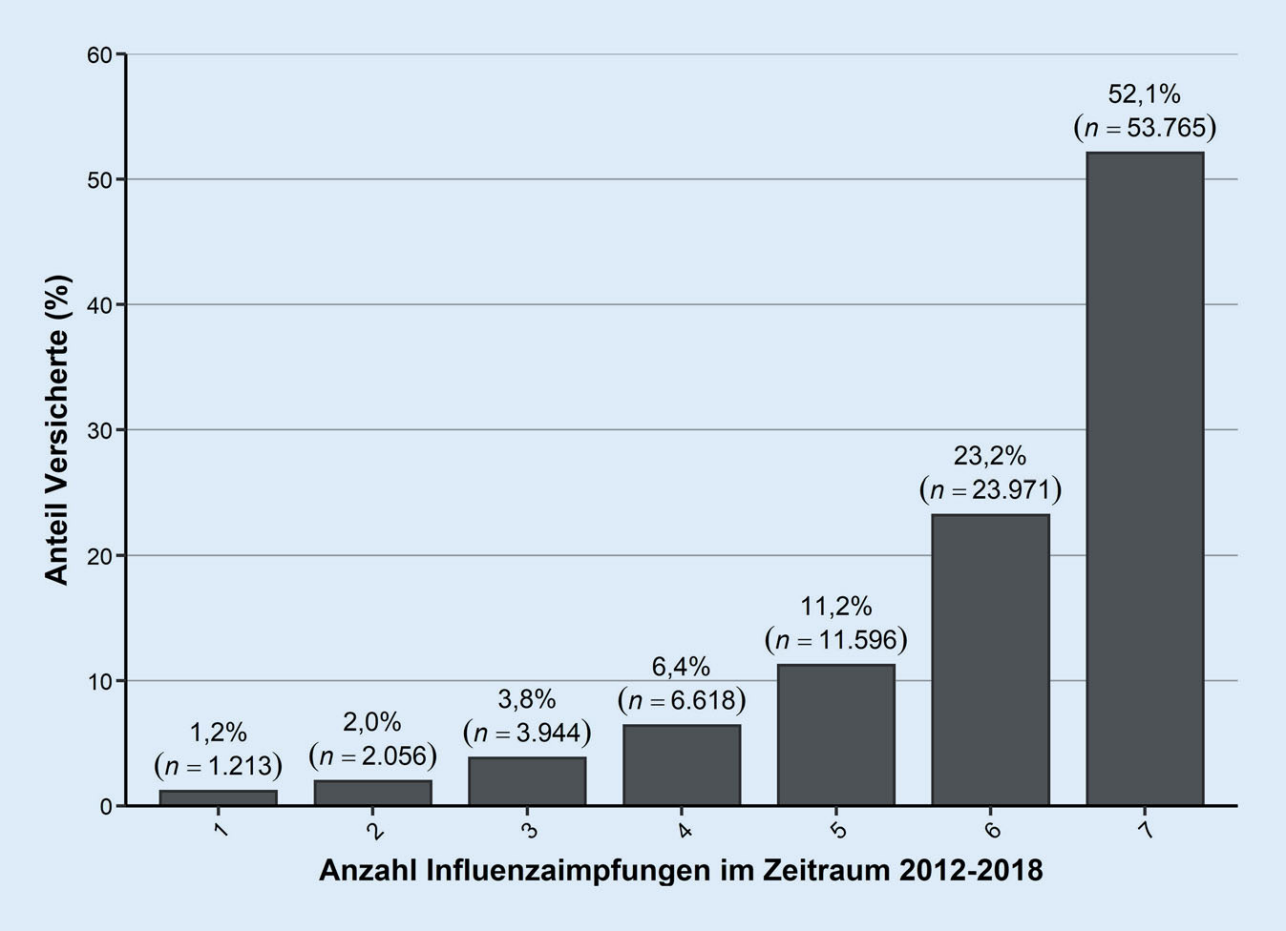

Von der Studienpopulation ließen sich in den 7 beobachteten Saisons 1,2 % (*n* *=* 1213) der Versicherten nur einmal gegen Influenza impfen, während sich weitere 52,1 % (*n* *=* 53.765) in allen 7 Saisons impfen ließen (Abb. 1). 23,2 % (*n* *=* 23.971) der Versicherten ließen sich in den 7 Saisons 6‑mal impfen.

Von maximal 7 Impfungen wurden durchschnittlich 5,9 (Median 6,0) durch einen Hausarzt/eine Hausärztin und nicht durch eine andere niedergelassene Fachärzt:in abgerechnet (Tab. 1). Dies entspricht einem Anteil von 84,4 %. Durchschnittlich 5,5 Influenzaimpfungen (Median 6,0) wurden durch dieselbe Arztpraxis abgerechnet. Bezogen auf diejenigen Versicherten, die mindestens 2 Impfungen erhalten haben, entspricht das einem Anteil von 78,3 %.

### Prädiktoren der Anzahl der Influenzaimpfungen

In dem multiplen Modell zeigte sich ein statistisch signifikanter Zusammenhang zwischen den Altersgruppen und der Anzahl der Impfungen. Verglichen mit der Altersgruppe der 60- bis 69-Jährigen ergab sich, kontrolliert für den Effekt aller anderen Prädiktoren, eine RR von 1,17 (95 %-KI 1,14; 1,19) für die Altersgruppe der 70- bis 79-Jährigen (Tab. 2). Die Anzahl der Impfungen war somit in dieser Altersgruppe um 17 % höher als bei den 60- bis 69-Jährigen. Bei den 80- bis 89‑Jährigen war sie um 13 % höher (RR 1,13; 95 %-KI 1,10; 1,16) und bei den ≥ 90-Jährigen um 25 % höher (RR 1,25; 95 %-KI 1,11; 1,40) als in der Altersgruppe der 60- bis 69‑Jährigen. Bei den Frauen unserer Studienpopulation war die Zahl der Influenzaimpfungen um 9 % niedriger als bei den Männern (RR 0,91; 95 %-KI 0,90; 0,93).

**Table 2:** 

	Rate Ratio	95 %-KI	*p*-Wert
*Altersgruppe* (Referenz: 60–69 Jahre)			
70–79 Jahre	1,17*	1,14; 1,19	< 0,001
80–89 Jahre	1,13*	1,10; 1,16	< 0,001
≥ 90 Jahre	1,25*	1,11; 1,40	< 0,001
*Geschlecht* (Referenz: männlich)			
Weiblich	0,91*	0,90; 0,93	< 0,001
*Pflegestufe zum 31.12.2013*			
Pflegestufe 1 (inkl. Pflegestufe 0) vs. keine Pflegestufe	0,90*	0,86; 0,93	< 0,001
Pflegestufe 2 vs. Pflegestufe 1	0,95	0,89; 1,02	0,173
Pflegestufe 3 (inkl. Härtefälle) vs. Pflegestufe 2	0,98	0,85; 1,14	0,828
*Pflegeheim im Zeitraum 2012–2018 (Referenz: durchgängig nicht im Pflegeheim lebend)*			
Durchgängig im Pflegeheim lebend	1,27*	1,18; 1,37	< 0,001
Mischform^a^	0,78	0,76; 0,81	< 0,001
*DMP-Teilnahme*			
Jahre mit DMP-Teilnahme	1,03*	1,02; 1,03	< 0,001
*Personen mit erhöhter gesundheitlicher Gefährdung im Jahr 2013* (Referenz: Personen ohne erhöhte gesundheitliche Gefährdung)			
Personen mit erhöhter gesundheitlicher Gefährdung	1,21*	1,17; 1,26	< 0,001
*Summe der Charlson-Komorbiditäten des Charlson Comorbidity Index (CCI) im Jahr 2013* (Referenz: CCI: 0)			
CCI: 1	0,97*	0,94; 0,999	0,044
CCI: 2–4	0,998	0,97; 1,03	0,911
CCI: ≥ 5	0,97	0,93; 1,004	0,079

Das negativ-binomiale Regressionsmodell war signifikant (Χ^2^[14] = 981,73; *p* < 0,001, Akaike-Information-Criterion = 281.078, Bayesian-Information-Criterion = 281.231) und erklärt die Daten signifikant besser als ein Poisson-Modell (Likelihood Ratio Test Χ^2^[1] = 23.692,28, *p* < 0,001)

*DMP* Disease-Management-Programm, *KI* Konfidenzintervall, *SE* Standardfehler

^a^ Um eine Reduktion der Teststärke durch Fallausschluss zu verhindern und im Regressionsmodell die gesamte Studienpopulation betrachten zu können, gingen diese Versicherten ins Regressionsmodell ein. Auf eine Interpretation der Ergebnisse dieser Gruppe wird aufgrund ihrer heterogenen Zusammensetzung allerdings verzichtet

*p-Wert < 0,05

Eine statistisch signifikante Reduktion der Anzahl der Influenzaimpfungen zeigte sich bei Versicherten mit PflS 1 (inkl. PflS 0). Diese war um 10 % niedriger als bei Versicherten ohne PflS (RR 0,90; 95 %-KI 0,86; 0,93). Versicherte, die im Beobachtungszeitraum durchgängig im Pflegeheim lebten, hatten eine um 27 % höhere Anzahl von Influenzaimpfungen als Versicherte, die nicht im Pflegeheim lebten (RR 1,27; 95 %-KI 1,18; 1,37).

Mit jedem zusätzlichen Jahr der DMP-Teilnahme stieg die Anzahl der Influenzaimpfungen um 3 % (RR 1,03; 95 %-KI 1,02; 1,03).

Bei Personen mit erhöhter gesundheitlicher Gefährdung war die Anzahl der Impfungen um 21 % höher als bei Versicherten ohne eine dieser zugrunde liegenden Erkrankungen (RR 1,21 %; 95 %-KI 1,17; 1,26). Hinsichtlich der Komorbidität zeigte sich ein statistisch signifikantes Ergebnis nur bei Versicherten mit einer CCI-Komorbidität. Hier war die Zahl der Impfungen um 3 % niedriger als bei Versicherten ohne CCI-Komorbidität (RR 0,97; 95 %-KI 0,94; 0,999).

## Diskussion

### Anzahl der Influenzaimpfung

Mehr als drei Viertel der eingeschlossenen Versicherten ließen sich in mindestens 6 von 7 beobachteten Saisons gegen Influenza impfen und werden als regelmäßig geimpft betrachtet. Mehr als 80 % der Impfungen wurden durch Hausärzt:innen und ebenso fast 80 % der Influenzaimpfungen bei Versicherten mit mindestens 2 Impfungen durch dieselbe Praxis abgerechnet. Dies entspricht der grundsätzlich bekannten Versorgungsrealität, dass insbesondere Hausärzt:innen gegen Influenza impfen [22, 23, 28]. Ihnen kommt zudem vor allem in Pflegeeinrichtungen eine zentrale Rolle zu. So kamen Spreckelsen et al. zu dem Ergebnis, dass mehr als 97 % der Pflegeheimbewohner:innen durch Hausärzt:innen gegen Influenza geimpft werden [23]. Kurch-Bek et al. zeigten, dass die Impfquoten von Pflegeheimbewohner:innen bei Inanspruchnahme hausärztlicher Versorgung höher lagen als ohne Inanspruchnahme [22].

In unserer Studie liegt der Anteil regelmäßig Geimpfter über dem Anteil, der trotz vergleichbarer Impfempfehlungen [12, 29] in anderen Untersuchungen ermittelt wurde. Internationale Studien zeigen ein heterogenes Bild: 58,6 % der ≥ 65-Jährigen in einer US-amerikanischen Befragungsstudie von Bardenheier et al. gaben an, in den Jahren 2004–2016 regelmäßig gegen Influenza geimpft worden zu sein [10]. Zu vergleichbaren Ergebnissen kam eine US-amerikanische Registerdatenstudie von McLean et al., in der sich mehr als die Hälfte der ≥ 60-Jährigen regelmäßig gegen Influenza impfen ließ [11]. Hingegen kam eine taiwanesische Untersuchung von Hsu et al. auf Basis von Versicherungsdaten zu dem Ergebnis, dass sich nur 32,7 % bzw. 24,9 % der Geimpften in 6 bzw. 7 aufeinanderfolgenden Jahren gegen Influenza impfen ließen [12], was deutlich unter dem hier gezeigten Anteil regelmäßig Geimpfter liegt. In einer deutschlandweiten Studie auf Basis von KV-Daten zeigten Reuss et al., dass sich 48 % der ≥ 60‑Jährigen in allen 3 betrachteten Saisons 2004/2005–2006/2007 gegen Influenza impfen ließen [14].

Hauptgrund für den höheren Anteil regelmäßig Geimpfter in unserer Studienpopulation ist das Einschlusskriterium der Influenzaimpfung in 2014Q34, da sich Versicherte, die sich einmal gegen Influenza impfen ließen, dies dann anscheinend regelmäßig tun. Zudem sind die Influenzaimpfquoten in Ostdeutschland historisch bedingt höher als in Westdeutschland [30–32]. Daher ist es möglich, dass ebenfalls ein Ost-West-Unterschied hinsichtlich der wiederholten Impfung besteht. Ferner resultieren die Unterschiede im Anteil regelmäßig Geimpfter auch aus der fehlenden Definition von „regelmäßig geimpft“. So galten in der Studie von McLean et al. Personen in einem Beobachtungszeitraum von 8 Saisons als regelmäßig geimpft, wenn sie in den vergangenen 5 Saisons 4–5 Influenzaimpfungen erhalten hatten [11]. Hingegen galten Versicherte in der Untersuchung von Hsu et al. dann als regelmäßig geimpft, wenn sie sich ab dem Zeitpunkt des Studieneinschlusses ohne Unterbrechung jährlich impfen ließen [12]. Schließlich ist aufgrund unterschiedlicher Rahmenbedingungen (Gesundheitssystem, nationale Impfempfehlungen), Studiendesigns (Datenbasis, Einschlusskriterien) und betrachteter Saisons von 1999/2000 bis 2012/2013 (zirkulierende Viren, Effektivität der saisonalen Impfstoffe) die Vergleichbarkeit mit internationalen Studien begrenzt.

### Prädiktoren der Anzahl von Influenzaimpfungen

Wir konnten zeigen, dass ≥ 60-Jährige mit zunehmendem Alter häufiger geimpft sind. Dieses Ergebnis entspricht sowohl dem anderer Studien zur wiederholten Influenzaimpfung [10, 20] als auch Studien mit Querschnittserhebung der Impfquoten, in denen mit zunehmendem Alter auch die Zahl der Influenzageimpften stieg [22, 26–28, 33].

In unserer Untersuchung waren Frauen weniger häufig wiederholt geimpft als Männer. Zu vergleichbaren Ergebnissen kamen auch Querschnittserhebungen auf Basis von Abrechnungsdaten von Sawicki et al. und Dios-Guerra et al. [26, 34]. Allerdings weist die Mehrzahl der Studien auf grundsätzlich höhere Impfquoten [21, 22, 27, 35] und auch regelmäßigere Influenzaimpfungen bei Frauen hin [10, 11, 14]. Eine mögliche Erklärung für unsere davon abweichenden Ergebnisse könnte sein, dass dieser Geschlechtereffekt zugunsten von Frauen in höheren Altersgruppen abnimmt [10] und Männer, die sich einmal gegen Influenza impfen lassen, dies im Alter dann auch häufiger wiederholt tun.

Ferner konnten wir zeigen, dass Versicherte mit PflS 1 weniger häufig geimpft waren als Versicherte ohne PflS. Dieser Vergleich war in unserer Studienpopulation nur bei in der Häuslichkeit lebenden Versicherten möglich, da es keine im Pflegeheim lebenden Versicherte ohne PflS gab. Somit könnte dies ein Hinweis darauf sein, dass in der Häuslichkeit Lebende mit Pflegebedarf hinsichtlich der Influenzaimpfung schlechter versorgt sind als Personen ohne Pflegebedarf. Dieses Phänomen ließe sich auf den *Healthy Adherer Effect* zurückführen, nach dem Ältere mit weniger funktionellen Einschränkungen selbst einen Arzt aufsuchen können und somit häufiger geimpft sind als Ältere mit Einschränkungen [36, 37].

Pflegeheimbewohner:innen waren deutlich häufiger geimpft als Nichtpflegeheimbewohner:innen. Kongruent dazu zeigten andere Studien häufigere Impfungen bei Pflegeheimbewohner:innen [27] und steigende Impfraten bei Einzug ins Pflegeheim [23]. Global lassen sich diese Ergebnisse auf die Influenzaimpfempfehlung für Pflegeheimbewohner:innen zurückführen [7, 38]. Deren Umsetzung erfolgt jedoch nicht in allen Pflegeeinrichtungen gleichmäßig. Einige Pflegeheime führen Impfprogramme durch, bei denen Impfindikationen und deren Umsetzung standardisiert geprüft werden, um Impflücken zu schließen [39]. Aufgrund der Struktur der vorliegenden Daten konnten wir derartige Zentrumseffekte jedoch nicht empirisch belegen.

Versicherte, die in mindestens ein DMP eigeschrieben waren, sind etwas häufiger gegen Influenza geimpft. Ein Hauptgrund hierfür könnte sein, dass überwiegend Hausärzt:innen die DMP-Versorgung und Impfung übernehmen. So ermöglicht die Teilnahme eines oder einer Versicherten an einem DMP durch Versorgungskontinuität regelmäßige nichtakute ärztliche Kontakte und bietet folglich häufigere Gelegenheiten für eine Influenzaimpfung.

Ebenfalls häufiger geimpft waren Personen mit erhöhter gesundheitlicher Gefährdung. Dieses Ergebnis steht im Einklang mit den geltenden Impfempfehlungen, aber auch mit dem Ergebnis von McLean et al., dass Personen, für die ein besonders hohes Erkrankungsrisiko besteht, sich regelmäßiger impfen lassen [11]. Ein Grund für den im Vergleich zu McLean et al. höheren Anteil regelmäßig Geimpfter in unserer Studie ist, dass für die von uns berücksichtigten Erkrankungen die Impfung explizit empfohlen wird [7].

In der Literatur finden sich Hinweise darauf, dass chronisch Kranke häufiger geimpft sind [26, 27, 40]. Unsere Studie, die einen multiplen Analyseansatz verfolgte, zeigte hingegen, dass Versicherte mit CCI-Komorbiditäten – wenn auch nur in sehr geringerem Maße – seltener geimpft waren als Versicherte ohne Komorbidität. Der in der Literatur berichtete Effekt lässt sich also nicht replizieren, wenn man für die hier berücksichtigten Einflussgrößen kontrolliert. Insbesondere die erhöhte gesundheitliche Gefährdung scheint bedeutsam zu sein, was eine explorative Anschlussanalyse mit einem Regressionsmodell ohne diesen Prädiktor nahelegt: Wird nicht für Personen mit erhöhter gesundheitlicher Gefährdung kontrolliert, so waren Versicherte mit einer bzw. 2–4 CCI-Komorbiditäten häufiger geimpft als Versicherte ohne Komorbidität (CCI 1: RR 1,03 (95 %-KI 1,001; 1,06) bzw. CCI 2–4: RR 1,06 (95 %-KI 1,03; 1,09)). Über die erhöhte gesundheitliche Gefährdung hinaus hatte die Anzahl der CCI-Komorbiditäten in unserer Untersuchung somit keinen zusätzlichen Erklärungswert für die häufigere Impfung.

### Stärken und Limitationen

Die vorliegende Routinedatenanalyse bildet erstmals das individuelle Influenzaimpfverhalten von ≥ 60-Jährigen in Deutschland ab. In der Population der bei der Thüringer AOK Plus Versicherten wurde die wiederholte Influenzaimpfung im Langzeitverlauf über 7 Saisons untersucht. Die breite Datenbasis ermöglichte es, unterschiedliche impfrelevante Versichertenmerkmale zu identifizieren und in den Analysen zu berücksichtigen.

Bei Krankenkassenabrechnungsdaten handelt es sich um administrative Daten, die zum Zweck der ärztlichen Honorarabrechnungen erhoben werden. So könnte die Anzahl der identifizierten Impfungen unterschätzt sein, da nur Daten niedergelassener Ärzt:innen zur Verfügung standen und beispielsweise die durch Betriebsärzt:innen erbrachten Impfungen nicht enthalten sind. Dies ist in der hier betrachteten Studienpopulation ≥ 60-Jähriger mit einem Anteil von Berufstätigen unter 4 % zu vernachlässigen. Zusätzlich handelt es sich bei dieser Arbeit um eine Sekundäranalyse einer Studienpopulation, in der alle betrachteten Versicherten mindestens einmal (in 2014Q34) gegen Influenza geimpft wurden. Dies hat zur Folge, dass Ungeimpfte bzw. Versicherte, die sich erstmalig nach der Saison 2014/2015 gegen Influenza impfen ließen, nicht analysiert werden konnten. Folglich führt dies zu einer höheren Anzahl von Wiederholungsimpfungen, als es in einer unselektierten Kohorte der Fall gewesen wäre, und kann somit eine Überschätzung der Anzahl der Influenzaimpfungen zur Folge haben. Zudem lagen die Daten für die Saison 2018/2019 nicht vollständig vor. Das könnte dazu führen, dass Versicherte, die sich vorranging im ersten Quartal eines Jahres gegen Influenza impfen lassen, unterschätzt werden und dann nur maximal 6 Impfungen gehabt haben können. Das Risiko ist als gering einzuschätzen, da sich Versicherte mit zunehmendem Alter überwiegend im dritten Quartal impfen lassen [33] und in unseren Daten > 80 % der Impfungen im dritten oder vierten Quartal abgerechnet wurden. Aufgrund des Stichtagsbezugs der PflS konnte eine Veränderung der PflS in den Analysen nicht berücksichtigt werden. So bleibt unklar, ob eine Erhöhung der PflS in Zusammenhang mit häufigeren Influenzaimpfungen steht.

Gleichzeitig können über Abrechnungsdaten nicht alle impfrelevanten Versichertenmerkmale abgebildet werden. Das führt dazu, dass beispielsweise der Einfluss von Gesundheitsverhalten, funktionellem Status oder der subjektiven Einstellung zur Influenzaimpfung auf die wiederholte Impfung nicht untersucht werden konnten. So können verschiedene psychologische Aspekte das individuelle Impfverhalten beeinflussen. Betsch et al. beschreiben im „5C-Modell“ die Faktoren Confidence (Vertrauen), Complacency (Risikowahrnehmung), Constraints (Barrieren in der Ausführung), Calculation (Ausmaß der Informationssuche) und Collective Responsibility (Verantwortungsgefühl für die Gemeinschaft) als Gründe der Impfentscheidung [41, 42]. Bock et al. konnten zudem zeigen, dass Selbstwertgefühl und wahrgenommener Stress Einfluss auf die Regelmäßigkeit von Influenzaimpfungen haben [43]. Daneben hängt das Impfverhalten auch von den Eigenschaften und der Einstellung der behandelnden Ärzt:innen ab. So wurde in mehreren Studien gezeigt, dass Ärztinnen, die eigene Influenzaimpfung der Ärzt:in sowie die Überzeugung der Ärzt:in von der Wirksamkeit der Impfung und die Impfberatung einen positiven Einfluss auf die Impfentscheidung der Patient:innen haben [28, 44, 45]. Zudem können saisonale Effekte wie die Verfügbarkeit des Impfstoffes und die Passung mit den zirkulierenden Viren sowie die wahrgenommene Schwere der vorangegangenen Influenzasaisons als mögliche Einflussfaktoren auf eine wiederholte Influenzaimpfung nicht ausgeschlossen werden.

Das Ost-West-Gefälle der Influenzaimpfquoten [30–32] und die hohe Gesamtmorbidität der Thüringer GKV-Versicherten [46] können mit einer höheren Anzahl von Impfungen einhergehen. Die Übertragung der Ergebnisse auf andere Bundesländer ist daher begrenzt und zur Klärung dieser Fragestellung wäre eine deutschlandweite Studie mit GKV-Routinedaten notwendig.

Seit September 2021 wird die simultane Impfung gegen Influenza und Coronavirus Disease 2019 (COVID-19) empfohlen [47]. Nationale oder internationale Daten, wie viele Personen in der Saison 2021/2022 eine Koadministration der Influenza- und COVID-19-Impfung in Anspruch genommen haben, liegen zum aktuellen Zeitpunkt nicht vor. In einer US-amerikanischen Studie konnte jedoch gezeigt werden, dass bei Personen, die sich regelmäßig gegen Influenza impfen lassen, eine sehr hohe Akzeptanz bezüglich der Koadministration besteht [48]. Zudem ist seit der Saison 2018/2019 ein leichter Anstieg der Influenzaimpfquote bei ≥ 60-Jährigen zu beobachten, welcher mit Beginn der COVID-19-Pandemie in der Saison 2020/2021 nochmals deutlich zunahm [49], in der Saison 2021/2022 aber wieder abnahm [50]. Daher ist davon auszugehen, dass der Anteil wiederholt Geimpfter mit Beginn der COVID-19-Pandemie ebenfalls gestiegen sein könnte und vor allem in vulnerablen Gruppen (z. B. Personen mit erhöhter gesundheitlicher Gefährdung) noch höher ist, mit dem Rückgang der Pandemie jedoch auch zurückgegangen sein kann.

## Fazit

Anhand von GKV-Routinedaten konnten wir über einen Zeitraum von 7 Jahren zeigen, dass mehr als drei Viertel der ≥ 60‑Jährigen Thüringer:innen, die sich einmal gegen Influenza impfen ließen, dies dann auch regelmäßig tun. Hausärzt:innen spielen dabei eine zentrale Rolle, da sie den Großteil der Impfungen übernehmen. Bei Versicherten mit erhöhter Versorgungskontinuität (beispielsweise bei DMP-Teilnahme) und den damit verbundenen regelmäßigen ärztlichen Vorstellungen ist die Zahl der Influenzaimpfungen höher. Ab dem 60. Lebensjahr sollten nichtakute ärztliche Kontakte konsequent genutzt werden, um die saisonale Influenzaimpfung anzubieten und Versicherte in ihrer Impfentscheidung zu unterstützen. Auch können professionell Pflegende und medizinische Fachangestellte dazu beitragen, die Regelmäßigkeit der Impfung positiv zu beeinflussen. Die von uns identifizierte, vergleichsweise geringere wiederholte Influenzaimpfung bei Frauen und Versicherten in häuslicher Pflege ist bemerkenswert und sollte zukünftig verstärkt in den Blick genommen werden.

## Supplementary Information




