# Supplementary material for: Once vaccinated, always vaccinated? A claims data analysis on repeated influenza vaccinations among individuals aged ≥ 60 years
Source: Bundesgesundheitsblatt Gesundheitsforschung Gesundheitsschutz. 2023 Jun 8;66(9):953–61. [Article in German] doi: 10.1007/s00103-023-03716-1 (PMC10249936; doi:10.1007/s00103-023-03716-1)
Supplement: Supplementary file 1 [file 103_2023_3716_MOESM1_ESM.pdf]

Onlinematerial

Einmal geimpft, immer geimpft?

Routinedatenanalyse zur wiederholten Influenzaimpfung von ≥60-Jährigen

Once vaccinated, always vaccinated?

A claims data analysis on repeated influenza vaccinations among individuals aged ≥ 60 years

Josephine Storch[1, 2], Franziska Meissner[1], Monique Böde[1], Anja Kwetkat[3], Mathias W. Pletz[4] & Antje Freytag[1]

[1] Universitätsklinikum Jena, Institut für Allgemeinmedizin, Friedrich-Schiller-Universität, Jena, Deutschland

[2] Internationale Graduierten Akademie, Medizinische Fakultät, Institut für Gesundheits- und Pflegewissenschaft, Martin-Luther-Universität Halle-Wittenberg, Halle (Saale), Deutschland

[3] Klinik für Geriatrie und Palliativmedizin, Klinikum Osnabrück, Osnabrück, Deutschland Klinik für Geriatrie, Universitätsklinikum Jena, Jena, Deutschland

[4] Universitätsklinikum Jena, Institut für Infektionsmedizin und Krankenhaushygiene, Friedrich-Schiller- Universität, Jena, Deutschland

Korrespondenzadresse:

Josephine Storch (M. Sc.)

Bachstraße 18

07743 Jena

josephine.storch@med.uni-jena.de

Übersicht

Primärstudie .... Seite 3

Abb. A1: Flowchart zum Versicherteneinschluss .... Seite 4

Datenaufbereitung: Identifikation von Inanspruchnahme- und Versichertenerkmalen .... Seite 5

Tab. A1: Impfungen .... Seite 5

Tab. A2: Personen mit erhöhter gesundheitlicher Gefährdung .... Seite 6

Tab. A3: Charlson-Komorbiditäten des Charlson Comorbidity-Index (CCI) .... Seite 7

Ergebnisse .... Seite 10

Tab. A4: Beschreibung der Studienpopulation, differenziert nach Anzahl der Influenza-impfungen .... Seite 10

Literatur .... Seite 14

Methoden

Primärstudie

Die Primärstudie [1, 2] erhielt von der Ethikkommission des Universitätsklinikum Jena ein positives Votum (Bearbeitungsnummer 4988-11/16) sowie die Genehmigung zur Übermittlung von Sozialdaten für die Forschung und Planung gemäß §75 SGB X durch das Sächsische Staatsministerium für Soziales und Verbraucherschutz [1, 2].

Für die Primärstudie wurden Daten derjenigen Versicherten übermittelt, die zum 01.01.2014 mindestens 60 Jahre alt waren, im Zeitraum 2008–2016 durchgängig bei der AOK Plus versichert waren, ihren Wohnsitz in Thüringen hatten oder nach 2014 verstorben waren und in 2014Q34 eine Influenzaimpfung und/oder im Jahr 2014 eine Pneumokokkenimpfung oder keine der beiden Impfungen im gesamten Beobachtungszeitraum erhalten hatten (*n* = 209.703, siehe Abb. A1) [1, 2].

Von der Studienpopulation der Primärstudie konnten nur die Versicherten in die Sekundäranalyse eingehen, die eine Influenzaimpfung oder eine Influenza- und Pneumokokkenimpfung erhalten hatten. Ungeimpfte Versicherte konnten nicht berücksichtigt werden, da diese per Definition im gesamten Beobachtungszeitraum der Sekundäranalyse (2012–2018) keine Influenzaimpfung erhalten hatten. Zur Minimierung eines Selektionsbias, wurden zudem Versicherte, die im Jahr 2014 ausschließlich eine Pneumokokkenimpfung erhalten hatten, nicht in der Sekundäranalyse berücksichtigt. Denn nur Versicherte, die 2014 ausschließlich gegen Pneumokokken geimpft wurden, können 2014 eine Influenzaimpflücke haben. Andere Gründe für eine Impflücke im Jahr 2014 wären dann – im Vergleich zu Impflücken in allen anderen Jahren – unterrepräsentiert. Somit haben alle Versicherten der Ausgangspopulation mindestens im Jahr 2014 eine Influenzaimpfung erhalten und können Impflücken in allen anderen Jahren des Beobachtungszeitraums haben. Insgesamt gingen die Daten von 142.022 Versicherten in die Ausgangspopulation der Sekundäranalyse ein (siehe Abb. A1).

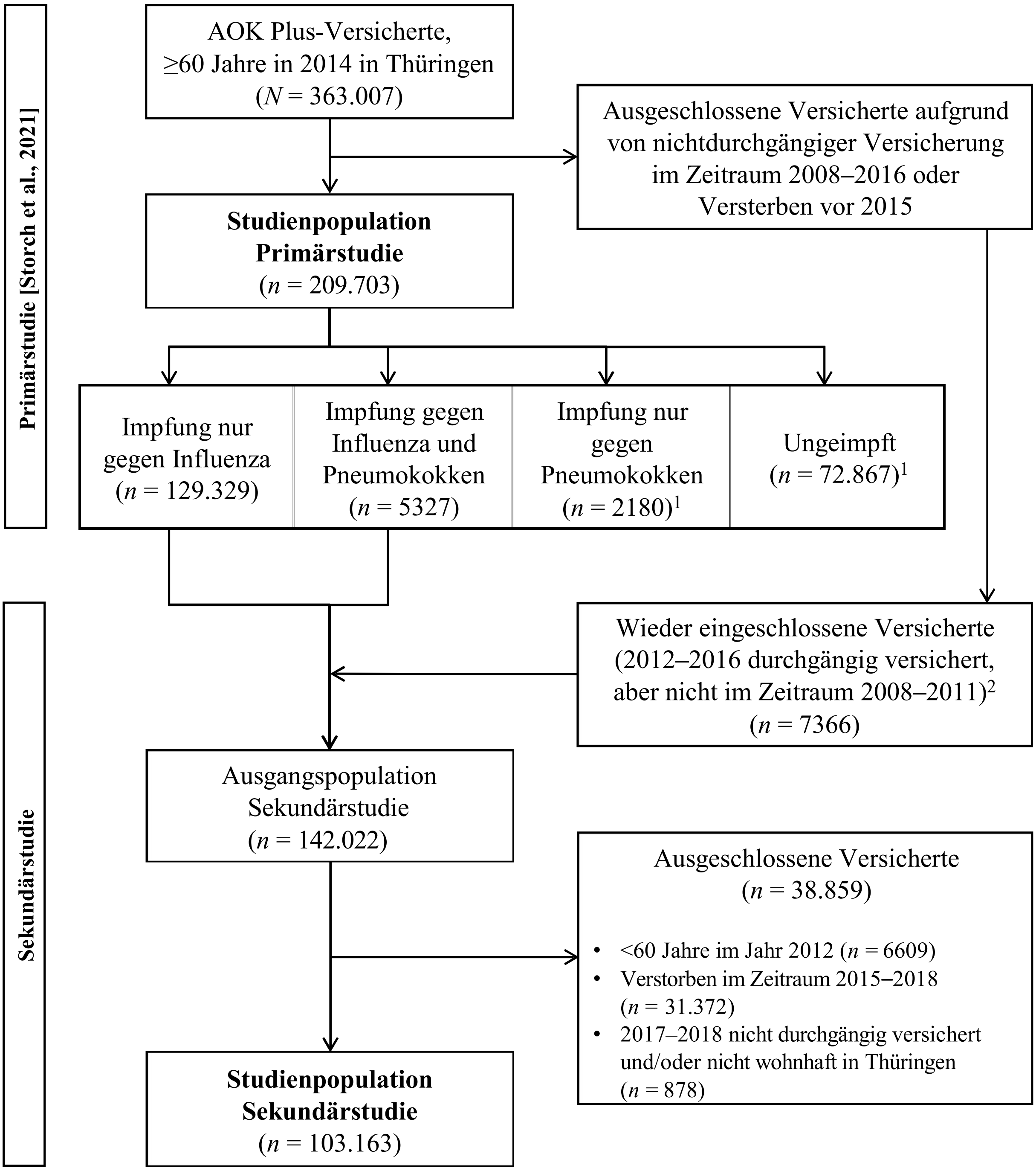

[1] Zur Minimierung eines Selektionsbias gingen Versicherte der Primärstudie, die ungeimpft oder nur gegen Pneumokokken geimpft waren, nicht in die Sekundärstudie ein.

[2] In der Primärstudie galt in allen Gruppen die durchgängige Versicherung im Zeitraum 2008-2016 als Einschlusskriterium. Für die Sekundärstudie kann diese Einschlussbedingung gelockert werden, indem Versicherte mit Influenzaimpfung, die 2012-2016 durchgängig und nur 2008-2011 nicht durchgängig bei der AOK Plus versichert waren, wieder eingeschlossen werden.

Abb. A1: Flowchart zum Versicherteneinschluss. (Eigene Abbildung)

Datenaufbereitung: Identifikation von Inanspruchnahme- und Versichertenmerkmalen

Tab. A1: Impfungen

Impfung	Gebührenordnungspositionen nach EBM (allgemeingültig und KVT-spezifisch)
Influenzaimpfung	89111, 89112, 89112 N, 89960, 89960 A, 89960 E, 89960 B, 89960 I, 89960 K
Pneumokokkenimpfung[1]	89118 A, 89118 B, 89119, 89120, 89120 R, 89964, 89964 A, 89964 E, 89964 B, 89964 I, 89964 K

EBM Einheitlicher Bewertungsmaßstab; **KVT** Kassenärztliche Vereinigung Thüringen

[1] Diese Gebührenordnungspositionen wurden zur Überprüfung des Ausschlusskriteriums einer Pneumokokken-Impfung herangezogen.

Tab. A2: Personen mit erhöhter gesundheitlicher Gefährdung[2]

Grunderkrankung	ICD-10-GM
Chronische Immunsuppression	M05, M06, M08, M30, M31, M32, M33, M34, M45, M120, M123, M350, M351, M352, M353, M354, M355, M356, M360, M461, M468, M469, K50, K51, L940, L941, L943 in Verbindung mit ATC: H01AB, H02B, L01XCO2, L04<br><br>B20, B21, B22, B23, B24, C00, C01, C02, C03, C04, C05, C06, C07, C08, C09, C10, C11, C12, C13, C14, C15, C16, C17, C18, C19, C20, C21, C22, C23, C24, C25, C26, C30, C31, C32, C33, C34, C37, C40, C41, C43, C45, C46, C47, C48, C49, C50, C51, C52, C53, C54, C55, C56, C57, C58, C60, C61, C62, C63, C64, C65, C66, C67, C68, C69, C70, C71, C72, C73, C74, C75, C76, C77, C78, C79, C80, C81, C82, C83, C84, C85, C88, C90, C91, C92, C93, C94, C95, C96, C97, D45, D46, D56, D57, D58, D59, D60, D61, D63, D65, D66, D67, D68, D69, D70, D71, D72, D74, D75, D76, D77, D80, D81, D82, D83, D84, D89, D90, D471, D473, D474, D475, D477, D479, D730, D731, D732, D735, D738, D739, Q890, Q899, U60, U61, U603, Z08, Z21, Z94, Z510, Z511, Z512
Herzerkrankung	A520, I05, I06, I07, I08, I09, I11, I12, I13, I15, I20, I21, I22, I23, I24, I25, I26, I27, I28, I34, I35, I36, I37, I38, I39, I42, I43, I47, I48, I49, I50, I51, I52, I71, I731, I738, I771, I790, I792, K551, K558, K559, P290, Q20, Q21, Q22, Q23, Q24, Q251, Z952, Z953, Z954, Z958, Z959
Lungenerkrankung	C34, D860, D862, E84, I278, I279, J40, J41, J42, J43, J44, J45, J46, J47, J60, J61, J62, J63, J64, J65, J66, J67, J68, J69, J70, J80, J81, J82, J84, J96, P27
Neurologische Erkrankung	G10, G11, G12, G13, G14, G20, G21, G22, G23, G24, G25, G26, G30, G31, G32, G35, G36, G37, G40, G41, G45, G46, G70, G71, G72, G73, G80, G81, G82, G832, G834, G839, G931, G934, H34, I60, I61, I62, I63, I64, I65, I66, I67, I68, I69, R56, R470
Nierenerkrankung	I12, I13, I701, I722, I823, N01, N02, N03, N04, N05, N06, N07, N08, N11, N12, N13, N14, N15, N16, N17, N18, N19, N250, Q60, Z49, Z940, Z992
Stoffwechselerkrankung	E03, E05, E10, E11, E12, E13, E14, E15, E16, E20, E21, E22, E25, E26, E27, E31, E32, E66, E70, E71, E72, E74, E75, E76, E77, E78, E79, E80, E83, E84, E85, E88, E89, E90, G3181, T383
Lebererkrankungen	B15, B16, B17, B18, B19, F100, F102, F105, F106, F107, F108, F109, I85, I864, I982, K70, K73, K74, K76, K711, K713, K714, K715, K717, K721, K727, K729, K750, K751, K754, K758, K759

[2] Definition basierend auf der Influenza-Indikationsimpfempfehlung für Kinder, Jugendliche und Erwachsene mit erhöhter gesundheitlicher Gefährdung infolge eines Grundleidens [3].

Tab. A3: Charlson-Komorbiditäten des Charlson Comorbidity-Index (CCI)[3]

Charlson-Komorbidität	ICD-10-GM
Myocardial infarction	I210, I211, I212, I213, I214, I219, I220, I221, I228, I229, I2520, I2521, I2522, I2529
Congestive heart failure	I099, I255, I420, I425, I426, I427, I429, I430, I431, I432, I438, I509, I1100, I1101, I1300, I1301, I1320, I1321, I4280, I4288, I5000, I5001, I5011, I5012, I5013, I5014, I5019, P290
Peripheral vascular disease	I700, I701, I708, I709, I711, I712, I713, I714, I715, I716, I718, I719, I731, I738, I739, I771, I790, I792, I7020, I7021, I7022, I7023, I7024, I7025, I7100, I7101, I7102, I7103, I7104, I7105, I7106, I7107, K551, K559, K5581, K5582, K5588, Z959, Z9580, Z9581, Z9588
Cerebrovascular disease	G460, G461, G462, G463, G464, G465, G466, G467, G468, G4502, G4503, G4509, G4512, G4513, G4519, G4522, G4523, G4529, G4532, G4533, G4539, G4542, G4543, G4549, G4582, G4583, G4589, G4592, G4593, G4599, H340, I64, I600, I601, I602, I603, I604, I605, I606, I607, I608, I609, I610, I611, I612, I613, I614, I615, I616, I618, I619, I621, I629,I630, I631, I632, I633, I634, I635, I636, I638, I639, I650, I651, I652, I653, I658, I659, I660, I661, I662, I663, I664, I668, I669, I670, I672, I673, I674, I675, I676, I677, I679, I680, I681, I682, I688, I690, I691, I692, I693, I694, I698, I6710, I6711, I6200, I6201, I6202, I6209, I6780, I6788
Dementia	F03, F010, F011, F012, F013, F018, F019, F020, F021, F022, F023, F024, F028, F051, F000, F001, F002, F009, G300, G301, G308, G309, G311
Chronic pulmonary disease	I278, I279, J40, J42, J46, J47, J60, J61, J64, J65, J410, J411, J418, J430, J431, J432, J438, J439, J450, J451, J458, J459, J620, J628, J630, J631, J632, J633, J634, J635, J638, J660, J661, J662, J668, J670, J671, J672, J673, J674. J675, J676, J677, J678, J679, J684, J701, J703, J4400, J4401, J4402, J4403, J4409, J4410, J4411, J4412, J4413, J4419, J4480, J4481, J4482, J4483, J4489, J4490, J4491, J4492, J4493, J4499
Rheumatic disease	M0500, M0501, M0502, M0503, M0504, M0505, M0506, M0507, M0508, M0509, M0510, M0511, M0512, M0513, M0514, M0515, M0516, M0517, M0518, M0519, M0520, M0521, M0522, M0523, M0524, M0525, M0526, M0527, M0528, M0529, M0530, M0531, M0532, M0533, M0534, M0535, M0536, M0537, M0538, M0539, M0580, M0581, M0582, M0583, M0584, M0585, M0586, M0587, M0588, M0589, M0590, M0591, M0592, M0593, M0594, M0595, M0596, M0597, M0598, M0599, M0600, M0601, M0602, M0603, M0604, M0605, M0606, M0607, M0608, M0609, M0610, M0611, M0612, M0613, M0614, M0615, M0616, M0617, M0618, M0619, M0620, M0621, M0622, M0623, M0624, M0625, M0626, M0627, M0628, M0629, M0630, M0631, M0632, M0633, M0634, M0635, M0636, M0637, M0638, M0639, M0640, M0641, M0642, M0643, M0644, M0645, M0646, M0647, M0648, M0649, M0680, M0681, M0682, M0683, M0684, M0685, M0686, M0687, M0688, M0689, M0690, M0691, M0692, M0693, M0694, M0695, M0696, M0697, M0698, M0699, M315, M320, M321, M328, M329, M330, M331, M332, M339, M340, M341, M342, M348, M349, M351, M353, M360
Peptic ulcer disease	K250, K251, K252, K253, K254, K255, K256, K257, K259, K260, K261, K262,

[3] Definition von Schwarzkopf et al. (2018) [4].

Charlson-Komorbidität	ICD-10-GM
	K263, K264, K265, K266, K267, K269, K270, K271, K272, K273, K274, K275, K276, K277, K279, K280, K281, K282, K283, K284, K285, K286, K287, K289
Mild liver disease	B180, B181, B182, B188, B189, K700, K701, K702, K703, K709, K713, K714, K715, K717, K730, K731, K732, K738, K739, K740, K741, K742, K743, K744, K745, K746, K760, K762, K763, K764, K768, K769, Z944
Diabetes without chronic complication	E1001, E1080, E1011, E1060, E1061, E1081, E1090, E1091, E1101, E1111, E1160, E1161, E1180, E1181, E1190, E1191, E1201, E1211, E1260, E1261, E1280, E1281, E1290, E1291, E1301, E1311, E1360, E1361, E1380, E1381, E1390, E1391, E1401, E1411, E1460, E1461, E1480, E1481, E1490, E1491
Diabetes with chronic complication	E1020, E1021, E1030, E1031, E1040, E1041, E1050, E1051, E1072, E1073, E1074, E1075, E1120, E1121, E1130, E1131, E1140, E1141, E1150, E1151, E1172, E1173, E1174, E1175, E1220, E1221, E1230, E1231, E1240, E1241, E1250, E1251, E1272, E1273, E1274, E1275, E1320, E1321, E1330, E1331, E1340, E1341, E1350, E1351, E1372, E1373, E1374, E1375, E1420, E1421, E1430, E1431, E1440, E1441, E1450, E1451, E1472, E1473, E1474, E1475
Hemiplegia or paraplegia	G041, G114, G801, G802, G810, G811, G819, G830, G831, G832, G833, G839, G8200, G8201, G8202, G8203, G8209, G8210, G8211, G8212, G8213, G8219, G8220, G8221, G8222, G8223, G8229, G8230, G8231, G8232, G8233, G8239, G8240, G8241, G8242, G8243, G8249, G8250, G8251, G8252, G8253, G8259, G8260, G8261, G8262, G8263, G8264, G8265, G8266, G8267, G8269, G8340, G8341, G8349
Renal disease	I1200, I1201, I1310, I1311, N19, N032, N033, N034, N035, N036, N037, N052, N053, N054, N055, N056, N057, N181, N182, N183, N184, N185, N189, N250, N1880, N1889, Z490, Z491, Z492, Z940, Z992
Any malignancy	C000, C001, C002, C003, C004, C005, C006, C008, C009, C01, C020, C021, C022, C023, C024, C028, C029, C030, C031, C039, C040, C041, C048, C049, C050, C051, C052, C058, C059, C060, C061, C062, C068, C069, C07, C080, C081, C088, C089, C090, C091, C098, C099, C100, C101, C102, C103, C104, C108, C109, C110, C111, C112, C113, C118, C119, C12, C130, C131, C132, C138, C139, C140, C142, C148, C150, C151, C152, C153, C154, C155, C158, C159, C160, C161, C162, C163, C164, C165, C166, C168, C169, C170, C171, C172, C173, C178, C179, C180, C181, C182, C183, C184, C185, C186, C187, C188, C189, C19, C20, C210, C211, C212, C218, C220, C221, C222, C223, C224, C227, C229, C23, C240, C241, C248, C249, C250, C251, C252, C253, C254, C257, C258, C259, C260, C261, C268, C269, C300, C301, C310, C311, C312, C313, C318, C319, C320, C321, C322, C323, C328, C329, C33, C340, C341, C342, C343, C348, C349, C37, C380, C381, C382, C383, C384, C388, C390, C398, C399, C400, C401, C402, C403, C408, C409, C4101, C4102, C411, C412, C4130, C4131, C4132, C414, C418, C419, C430, C431, C432, C433, C434, C435, C436, C437, C438, C439, C450, C451, C452, C457, C459, C460, C461, C462, C463, C467, C468, C469, C470, C471, C472, C473, C474, C475, C476, C478, C479, C480, C481, C482, C488, C490, C491, C492, C493, C494, C495, C496, C498, C499, C500, C501, C502, C503, C504, C505, C506, C508, C509, C510, C511, C512, C518, C519, C52, C530, C531, C538, C539, C540, C541, C542, C543, C548, C549, C55, C56, C570, C571, C572, C573, C574, C577, C578, C579, C58, C600, C601, C602, C608, C609, C61, C620, C621, C629, C630, C631, C632, C637, C638, C639, C64, C65, C66, C670, C671, C672, C673, C674, C675, C676, C677, C678, C679, C680, C681, C688, C689, C690, C691, C692, C693, C694, C695, C696, C698, C699, C700, C701, C709, C710, C711, C712, C713, C714, C715, C716, C717, C718, C719,

Charlson-Komorbidität	ICD-10-GM
	C720, C721, C722, C723, C724, C725, C728, C729, C73, C740, C741, C749, C750, C751, C752, C753, C754, C755, C758, C759, C760, C761, C762, C763, C764, C765, C767, C768, C810, C811, C812, C813, C814, C817, C819, C820, C821, C822, C823, C824, C825, C826, C827, C829, C830, C831, C833, C835, C837, C838, C839, C840, C841, C844, C845, C846, C847, C848, C849, C851, C852, C857, C859, C860, C861, C862, C863, C864, C865, C866, C8800, C8801, C8820, C8821, C8830, C8831, C8840, C8841, C8870, C8871, C8890, C8891, C9000, C9001, C9010, C9011, C9020, C9021, C9030, C9031, C9100, C9101, C9110, C9111, C9130, C9131, C9140, C9141, C9150, C9151, C9160, C9161, C9170, C9171, C9180, C9181, C9190, C9191, C9200, C9201, C9210, C9211, C9220, C9221, C9230, C9231, C9240, C9241, C9250, C9251, C9260, C9261, C9270, C9271, C9280, C9281, C9290, C9291, C9300, C9301, C9310, C9311, C9330, C9331, C9370, C9371, C9390, C9391, C9400, C9401, C9420, C9421, C9430, C9431, C9440, C9441, C9460, C9461, C9470, C9471, C948, C9500, C9501, C9510, C9511, C9570, C9571, C958, C9590, C9591, C960, C962, C964, C965, C966, C967, C968, C969, C97
Moderate or severe liver disease	I850, I859, I864, I982, I983, K704, K711, K721, K729, K765, K766, K767
Metastatic solid tumor	C770, C771, C772, C773, C774, C775, C778, C779, C780, C781, C782, C783, C784, C785, C786, C787, C788, C790, C791, C792, C793, C794, C795, C796, C797, C799, C800, C809, C7981, C7982, C7983, C7984, C7988
AIDS/HIV	B20, B21, B22, B24

Ergebnisse

Tab. A4: Beschreibung der Studienpopulation, differenziert nach Anzahl der Influenzaimpfungen

	**Anzahl der Influenzaimpfungen 2012–2018**							
	**1**<br>*n* = 1.213<br>(1,2%)	**2**<br>*n* = 2.056<br>(2,0%)	**3**<br>*n* = 3.944<br>(3,8%)	**4**<br>*n* = 6.618<br>(6,4%)	**5**<br>*n* = 11.596<br>(11,2%)	**6**<br>*n* = 23.971<br>(23,2%)	**7**<br>*n* = 53.765<br>(52,1%)	**Gesamt**<br>*N* = 103.163
**Alter**								
**Alter (Jahre)**, MW (SD)	71,03<br>(7,2)	71,40<br>(7,4)	72,16<br>(7,4)	72,46<br>(7,3)	72,39<br>(7,3)	72,83<br>(7,1)	72,61<br>(6,8)	**72,57**<br>(7,0)
**Altersgruppe**, *n* (%)								
60–69 Jahre	520<br>(42,9%)	829<br>(40,3%)	1.440<br>(36,5%)	2.253<br>(34,0%)	4.050<br>(34,9%)	7.592<br>(31,7%)	17.005<br>(31,6%)	**33.689**<br>(32,7%)
70–79 Jahre	519<br>(42,8%)	905<br>(44,0%)	1.801<br>(45,7%)	3.076<br>(46,5%)	5.457<br>(47,1%)	11.902<br>(49,7%)	27.825<br>(51,8%)	**51.485**<br>(49,9%)
80–89 Jahre	170<br>(14,0%)	312<br>(15,2%)	675<br>(17,1%)	1.249<br>(18,9%)	2.016<br>(17,4%)	4.278<br>(17,8%)	8.615<br>(16,0%)	**17.315**<br>(16,8%)
≥ 90 Jahre	4<br>(0,3%)	10<br>(0,5%)	28<br>(0,7%)	40<br>(0,6%)	73<br>(0,6%)	199<br>(0,8%)	320<br>(0,6%)	**674**<br>(0,7%)
**Geschlecht**, *n* (%)								
männlich	490<br>(40,4%)	733<br>(35,7%)	1.383<br>(35,1%)	2.304<br>(34,8%)	4.285<br>(37,0%)	8.981<br>(37,5%)	21.951<br>(40,8%)	**40.127**<br>(38,9%)
weiblich	723<br>(59,6%)	1.323<br>(64,3%)	2.561<br>(64,9%)	4.314<br>(65,2%)	7.311<br>(63,0%)	14.990<br>(62,5%)	31.814<br>(59,2%)	**63.036**<br>(61,1%)

	Anzahl der Influenzaimpfungen 2012–2018							
	**1**<br>*n* = 1.213<br>(1,2%)	**2**<br>*n* = 2.056<br>(2,0%)	**3**<br>*n* = 3.944<br>(3,8%)	**4**<br>*n* = 6.618<br>(6,4%)	**5**<br>*n* = 11.596<br>(11,2%)	**6**<br>*n* = 23.971<br>(23,2%)	**7**<br>*n* = 53.765<br>(52,1%)	**Gesamt**<br>*N* = 103.163
**Pflegestufe zum 31.12.2013**, *n* (%)								
keine Pflegestufe	1.087<br>(89,6%)	1.853<br>(90,1%)	3.575<br>(90,6%)	5.937<br>(89,7%)	10.332<br>(89,1%)	21.576<br>(90,0%)	49.463<br>(92,0%)	**93.823**<br>(90,9%)
Pflegestufe 1 (inkl. Pflegestufe 0)	88<br>(7,3%)	135<br>(6,6%)	269<br>(6,8%)	507<br>(7,7%)	909<br>(7,8%)	1.693<br>(7,1%)	3.077<br>(5,7%)	**6.678**<br>(6,5%)
Pflegestufe 2	34<br>(2,8%)	59<br>(2,9%)	78<br>(2,0%)	151<br>(2,3%)	287<br>(2,5%)	601<br>(2,5%)	1.014<br>(1,9%)	**2.224**<br>(2,2%)
Pflegestufe 3 (inkl. Härtefälle)	4<br>(0,3%)	9<br>(0,4%)	22<br>(0,6%)	23<br>(0,3%)	68<br>(0,6%)	101<br>(0,4%)	211<br>(0,4%)	**438**<br>(0,4%)
**Pflegeheim im Zeitraum 2012–2018**, *n* (%)								
durchgängig nicht im Pflegeheim lebend	1.120<br>(92,3%)	1.855<br>(90,2%)	3.475<br>(88,1%)	5.754<br>(86,9%)	10.023<br>(86,4%)	21.191<br>(88,4%)	49.418<br>(91,9%)	**92.836**<br>(90,0%)
durchgängig im Pflegeheim lebend	13<br>(1,1%)	31<br>(1,5%)	61<br>(1,5%)	116<br>(1,8%)	223<br>(1,9%)	479<br>(2,0%)	1.115<br>(2,1%)	**2.038**<br>(2,0%)
Mischform	80<br>(6,6%)	170<br>(8,3%)	408<br>(10,3%)	748<br>(11,3%)	1.350<br>(11,6%)	2.301<br>(9,6%)	3.232<br>(6,0%)	**8.289**<br>(8,0%)
**DMP-Teilnahme im Zeitraum 2012–2018**								
Teilnahme an mind. 1 DMP, *n* (%)	687<br>(56,6%)	1.207<br>(58,7%)	2.358<br>(59,8%)	4.084<br>(61,7%)	7.352<br>(63,4%)	15.553<br>(64,9%)	34.467<br>(64,1%)	**65.708**<br>(63,7%)

	Anzahl der Influenzaimpfungen 2012–2018							
	**1**<br>*n* = 1.213<br>(1,2%)	**2**<br>*n* = 2.056<br>(2,0%)	**3**<br>*n* = 3.944<br>(3,8%)	**4**<br>*n* = 6.618<br>(6,4%)	**5**<br>*n* = 11.596<br>(11,2%)	**6**<br>*n* = 23.971<br>(23,2%)	**7**<br>*n* = 53.765<br>(52,1%)	**Gesamt**<br>*N* = 103.163
Jahre mit DMP-Teilnahme, MW (SD)	1,41<br>(1,8)	1,43<br>(1,8)	1,49<br>(1,8)	1,56<br>(1,8)	1,68<br>(1,9)	1,76<br>(1,9)	1,78<br>(2,0)	**1,73**<br>(1,9)
**Personen mit erhöhter gesundheitlicher Gefährdung (gem. STIKO-Impfempfehlung) im Jahr 2013**, *n* (%)								
**Personen mit erhöhter gesundheitlicher Gefährdung**	1.031<br>(85,0%)	1.821<br>(88,6%)	3.560<br>(90,3%)	6.104<br>(92,2%)	10.743<br>(92,6%)	22.484<br>(93,8%)	50.284<br>(93,5%)	**96.027**<br>(93,1%)
Chronische Immunsuppression	191<br>(15,7%)	325<br>(15,8%)	635<br>(16,1%)	1.110<br>(16,8%)	2.020<br>(17,4%)	4.509<br>(18,8%)	9.790<br>(18,2%)	**18.580**<br>(18,0%)
Herzerkrankung	604<br>(49,8%)	1.113<br>(54,1%)	2.191<br>(55,6%)	3.781<br>(57,1%)	6.843<br>(59,0%)	14.456<br>(60,3%)	32.111<br>(59,7%)	**61.099**<br>(59,2%)
Lungenerkrankung	288<br>(23,7%)	511<br>(24,9%)	1.011<br>(25,6%)	1.662<br>(25,1%)	2.925<br>(25,2%)	6.047<br>(25,2%)	12.961<br>(24,1%)	**25.405**<br>(24,6%)
Nierenerkrankung	200<br>(16,5%)	365<br>(17,8%)	745<br>(18,9%)	1.337<br>(20,2%)	2.408<br>(20,8%)	5.054<br>(21,1%)	10.853<br>(20,2%)	**20.962**<br>(20,3%)
Stoffwechselerkrankung	817<br>(67,4%)	1.464<br>(71,2%)	2.935<br>(74,4%)	5.043<br>(76,2%)	9.021<br>(77,8%)	18.922<br>(78,9%)	42.457<br>(79,0%)	**80.659**<br>(78,2%)
Neurologische Erkrankung	279<br>(23,0%)	493<br>(24,0%)	1.024<br>(26,0%)	1.762<br>(26,6%)	3.157<br>(27,2%)	6.626<br>(27,6%)	14.366<br>(26,7%)	**27.707**<br>(26,9%)
**Summe der Charlson-Komorbiditäten des Charlson Comorbidity-Index (CCI) im Jahr 2013**, *n* (%)								
CCI: 0	363<br>(29,9%)	531<br>(25,8%)	978<br>(24,8%)	1.453<br>(22,0%)	2.429<br>(20,9%)	4.758<br>(19,8%)	11.350<br>(21,1%)	**21.862**<br>(21,2%)

	Anzahl der Influenzaimpfungen 2012–2018							
	**1**<br>*n* = 1.213<br>(1,2%)	**2**<br>*n* = 2.056<br>(2,0%)	**3**<br>*n* = 3.944<br>(3,8%)	**4**<br>*n* = 6.618<br>(6,4%)	**5**<br>*n* = 11.596<br>(11,2%)	**6**<br>*n* = 23.971<br>(23,2%)	**7**<br>*n* = 53.765<br>(52,1%)	**Gesamt**<br>*N* = 103.163
CCI: 1	336<br>(27,7%)	522<br>(25,4%)	1.025<br>(26,0%)	1.737<br>(26,2%)	2.915<br>(25,1%)	5.953<br>(24,8%)	13.572<br>(25,2%)	**26.060**<br>(25,3%)
CCI: 2–4	420<br>(34,6%)	824<br>(40,1%)	1.592<br>(40,4%)	2.761<br>(41,7%)	5.111<br>(44,1%)	10.749<br>(44,8%)	23.663<br>(44,0%)	**45.120**<br>(43,7%)
CCI: ≥ 5	94<br>(7,7%)	179<br>(8,7%)	349<br>(8,8%)	667<br>(10,1%)	1.141<br>(9,8%)	2.511<br>(10,5%)	5.180<br>(9,6%)	**10.121**<br>(9,8%)
**Anzahl der Influenzaimpfungen pro Patient:in abgerechnet durch…** , MW (SD)								
einen Hausarzt/eine Hausärztin	0,97<br>(0,2)	1,93<br>(0,3)	2,90<br>(0,4)	3,91<br>(0,5)	4,89<br>(0,6)	5,88<br>(0,6)	6,88<br>(0,7)	**5,91**<br>(1,5)
dieselbe Praxis	1,00<br>(0,0)	1,84<br>(0,4)	2,76<br>(0,5)	3,64<br>(0,7)	4,51<br>(0,8)	5,41<br>(1,0)	6,39<br>(1,1)	**5,48**<br>(1,6)

DMP Disease-Management-Programm**; MW** Mittelwert; **SD** Standardabweichung; **STIKO** Ständige Impfkommission

Literatur

1. Storch J, Fleischmann-Struzek C, Rose N et al. (2021) The effect of influenza and pneumococcal vaccination in the elderly on health service utilisation and costs: a claims data-based cohort study. Eur J Health Econ 23:67–80. https://doi.org/10.1007/s10198-021-01343-8
2. Rose N, Storch J, Mikolajetz A et al. (2021) Preventive effects of influenza and pneumococcal vaccination in the elderly - results from a population-based retrospective cohort study. Hum Vaccin Immunother:1–9. https://doi.org/10.1080/21645515.2020.1845525
3. Robert Koch-Institut (2014) Empfehlungen der Ständigen Impfkommission (STIKO) am Robert Koch-Institut/Stand: August 2014. Epid Bull 34:305-340
4. Schwarzkopf D, Fleischmann-Struzek C, Rüddel H, Reinhart K, Thomas-Rüddel DO (2018) A risk- model for hospital mortality among patients with severe sepsis or septic shock based on German national administrative claims data. PLoS ONE 13:e0194371. https://doi.org/10.1371/journal.pone.0194371
